# The Immunological Mechanisms Involved in the Pathophysiology of Allergic Proctocolitis †

**DOI:** 10.3390/children12060688

**Published:** 2025-05-27

**Authors:** Jimena Pérez-Moreno, Esther Bernaldo-de-Quirós, Mar Tolín Hernani, Guillermo Álvarez-Calatayud, Laura Perezábad, César Sánchez Sánchez, Rafael Correa-Rocha

**Affiliations:** 1 Servicio de Pediatria, Digestivo Infantil, Hospital General Universitario Gregorio Marañón, 28007 Madrid, Spain; mariamar.tolin@salud.madrid.org (M.T.H.); guillermo.alvarez@salud.madrid.org (G.Á.-C.); cesar.sanchez.sanchez@salud.madrid.org (C.S.S.); rafael.correa@iisgm.com (R.C.-R.); 2 Laboratory of Immune-Regulation, Gregorio Marañón Health Research Institute, 28009 Madrid, Spain; esther.bernaldo@iisgm.com (E.B.-d.-Q.); lperezabad@bculinary.com (L.P.)

**Keywords:** cow’s milk protein allergy, non-IgE-mediated cow’s milk allergy, FPIAP, food allergy, regulatory T cells

## Abstract

Background: The pathophysiology of non-IgE-mediated cow’s milk allergy is mostly unknown. Previous studies suggested a mechanism mediated by T cells, but this was not confirmed in subsequent studies. The aim of this study was to investigate the immunological mechanisms, especially the role of regulatory T cells (Tregs), in the pathophysiology of allergic proctocolitis (FPIAP). Methods: A prospective observational study was conducted on infants with FPIAP and a control group of healthy infants with similar ages. The main variables were lymphocyte populations, included Tregs, which were extracted from peripheral blood and processed immediately by flow cytometry at two time points: in the acute phase (“T0”) and after clinical resolution (“Tres”). Results: A total of 32 patients with FPIAP and 10 healthy infants were enrolled. There was a higher T-CD4 memory cell count, increased numbers of regulatory B cells and a higher percentage of Tregs (*p* < 0.01) in patients with acute FPIAP in contrast to the healthy group. The levels of granulocytes (mainly eosinophils), dendritic cells (mDC2) and NK16+56- cells were also significantly higher in the FPIAP group. NK16+56- cells and the number of granulocytes appeared to be the best markers for distinguishing between the healthy and FPIAP infants based on the ROC curves. Conclusions: FPIAP does not appear to have an immune mechanism mediated by T cells, but it may be associated with innate immunity responses characterized by an increase in NK16+56- cells, eosinophils and dendritic cells. These cells could be evaluated in future studies as possible markers of non-IgE-mediated cow’s milk protein allergy.

## 1. Introduction

The pathophysiology of non-IgE-mediated cow’s milk protein allergy (CMPA) is mostly unknown despite its high prevalence (around 50% of pediatric food allergies) [1,2,3,4,5,6,7,8,9]. This explains the lack of diagnostic and predictive markers for this disorder [3].

CMPA is classified into different phenotypes: (i) IgE-mediated allergy, (ii) non-IgE-mediated allergy and (iii) combined IgE and cell-mediated allergy [4,5]. Current international and Spanish consensus guidelines classify non-IgE-mediated allergy into three main disorders: food protein-induced enterocolitis syndrome (FPIES), allergic proctocolitis (FPIAP) and food protein-induced enteropathy (FPE) [6,7,8,9,10,11]. FPIAP is a milder disorder and easy to diagnose based on the history of a healthy infant with streaks of blood and mucus in the stools that disappear after dietary avoidance without other manifestations [6,9]. The prevalence of FPIAP is about 0.2% in healthy infants but it is the cause of 64% of bloody stools in infants [12,13,14].

Unlike IgE-mediated CMPA, the symptoms of non-IgE CMPA are typically delayed from hours to weeks after protein ingestion and there is no confirmatory test available so the diagnosis could be a challenge. Previous studies in non-IgE-mediated cow’s milk allergy, especially the ones that study FPIES, suggested a mechanism mediated by T cells, but it was not confirmed in subsequent studies [15]. Treg cells are a subset of CD4+ T cells with a suppressive capacity, which is essential in promoting immune tolerance [16]. Some studies determined that the establishment of IgE-mediated CMPA was related to a deficit of Treg cells and a lower level of vitamin D that could have an influence on the preservation of the Treg population [17]. Therefore, an increase in Treg cells has been suggested as a marker of immunological tolerance after immunotherapy in FPIES [18].

In recent years, we have obtained more information about the natural history of FPIAP, the factors that influence this history and the involvement of the intestinal microbiota [19,20]. An immature microbiome (*Klebsiella* or *Shigella* as the dominant genus and a decreased abundance of *Bifidobacterium* and *Lactobacillus*) was found in symptomatic FPIAP infants [21]. Other studies reported alterations of specific cytokines (decreased levels of TGF-β, which is produced by regulatory T cells, and an increased level of proinflammatory cytokines like TNF-α), which could explain the increase in intestinal permeability and therefore the intestinal inflammation with bloody and mucus-streaked stools [22].

Despite the findings of previous studies and the guidelines published in recent years, the involvement of T cell and other cellular immune responses to food proteins in FPIAP has not been sufficiently studied. This could explain the heterogeneous management recommendations on the need for exclusion diet treatment due to the mild, benign, and transient nature of FPIAP and the high probability of spontaneous resolution [9]. Other treatments, like probiotics, have been proposed. *Lactobacillus rhamnosus* (*LGG*) supplemented in an extensively hydrolyzed formula has shown a beneficial effect on intestinal permeability and mucosal inflammation through enhancing the acquisition of tolerance [23].

The aim of this study was to investigate the role of Tregs and other immune mechanisms that are involved in the pathophysiology of FPIAP.

## 2. Materials and Methods

### 2.1. Patients and Study Enrolment

A prospective observational study was conducted in a tertiary hospital between January 2014 and January 2019. Infants with symptoms compatible with FPIAP were enrolled in the study at the Infant Gastroenterology Division of the hospital. The authors of the study were employees at the hospital, and they enrolled the infants, confirmed the diagnosis and collected blood samples. The confirmation of FPIAP was performed based on symptoms of rectal bleeding in a healthy infant without infection or other conditions that disappeared after dietary avoidance according to clinical guidelines [14] without colonoscopy or oral challenge. A control group of healthy infants with similar ages at the Pediatric Service who needed a blood sample for another condition (surgery or hyperbilirubinemia) were also enrolled. The study was conducted after approval by the ethics committee of the hospital and informed consent was obtained from all the parents before taking blood samples. The infants with FPIAP were divided in two groups: “T0-phase” infants in the acute phase that still have rectal bleeding and “Tres-phase” infants who showed clinical resolution after dietary avoidance. Clinical and analytical variables were collected until the infant acquired tolerance to cow’s milk protein after exposure to milk for 6–9 months, as recommended by the clinical guidelines.

### 2.2. Analysis of Immune Subsets and Treg Cells

The frequency and absolute counts of lymphocyte populations, including Treg cells, were analyzed in fresh peripheral blood by flow cytometry (Beckman Coulter, Villepinte, France) using a combination of specific antibodies, as previously described [24,25]. The sample processing and analysis of immune cells were performed by the Laboratory of Immune Regulation of the hospital using Kaluza software, version 2.1 (Beckman Coulter eBioscience, San Diego, CA, USA). Briefly, we analyzed CD4+T cells, CD8+T cells and subsets of T cells: naïve (CD45RA+CD27+), activated (HLA−DR+), central memory (CD45RA−CD27+) and effector memory (CD45RA−CD27−) cells. Tregs were analyzed without any washing steps that could modify the real values and were quantified by measuring CD3+CD4+CD25+CD127low cells and %Foxp3, which are markers that can better define the Treg phenotype. The percentage of Foxp3 cells was analyzed in PBMCs isolated in an Ficoll–Hypaque gradient using the Anti-Human Foxp3 Kit according to the manufacturer’s instructions (eBioscience, San Diego, CA, USA). We also measured the number of B cells (CD19+CD3−) including naïve (CD27−IgD+), memory non-switch (CD27+IgD+), memory switch (CD27+IgD−) and Breg (CD24highCD27high) phenotypes, and basophils (CD45lowCD123+IgE+) in whole blood samples. We analyzed cytokine-secreting CD4+T cells in isolated PBMCs after activation for 5 h with PMA (50 ng/mL) and ionomycin (1 ug/mL); Golgi stop was added and intracellular staining of IL-4, IFN-γ and IL-17 was performed using the Cytofix/Cytoperm Kit (Becton Dickinson, Franklin Lakes, NK). We also studied granulocytes, monocytes, natural killer (NK) cells and dendritic cells. The levels of 25-hydroxyvitamin D (ng/mL) were also quantified in serum samples (from FPIAP and control groups) by chemiluminescence using a LIASON XL analyzer.

### 2.3. Statistical Analysis

The statistical analysis was performed by the hospital’s Methodology and Biostatistics Unit using SPSS software version 21. Mann–Whitney and Kruskal–Wallis tests were used for comparing and correlating variables after using the Pearson correlation test. Rho Spearman was used to correlate clinical and immunological variables. ROC curves were generated using Stata program 8, SE version. A *p* value < 0.05 was considered significant.

## 3. Results

### 3.1. Clinical Characteristics of Enrolled Infants

We enrolled 32 patients with FPIAP (22 in *T*0 phase and 10 in *Tres* phase) and 10 infants in the control group. The sex and age distributions in the two groups were comparable, with a mean age of 2.47 ± 0.63 and 2.43 ± 0.29 months (*p* = 0.675) in the control and FPIAP groups, respectively.

In the FPIAP group, 65.5% were exclusively breastfed, 25% were on mixed lactation and 9.5% were completely bottle-fed. The total and specific IgE values of the plasma samples were negative. In the FPIAP group, 42% had a family history of atopy. Bloody stools were present in all patients, 25% had mucus in their stool, 15% had vomiting and 37.5% had abdominal pain. Different therapeutic strategies were adopted: 40.8% received extensive hydrolyzed formula without probiotics, 24.80% received extensive hydrolyzed formula with *Lactobacillus rhamnosus* and 34.4% went on a maternal elimination diet. No patients needed an elemental amino-acid formula or discontinuation of breast-feeding to achieve resolution of rectal bleeding.

The median time of symptom remission was 1 month (0.8–1.1). The mean time of reintroduction of cow milk protein was 10.3 ± 2 months. Both FPIAP groups (*T*0 and *Tres*) had similar symptoms and disease evolution during the clinical follow-up without significant differences.

### 3.2. Results from the Analysis of Immune Subsets and Treg Cells

**Figure 1:**
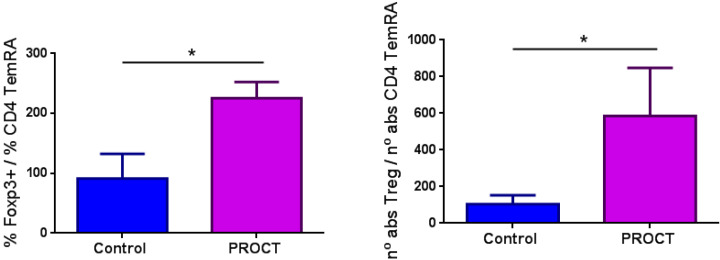
Treg(Foxp3)/TCD4+TemRA ratios in both study groups (PROCT (T0/FPIAP) and control groups). Foxp3 is a marker of Treg cells. TemRA: effector T cells; PROCT: FPIAP group. *: statistically significant.

There was no marked difference between the percentages and absolute counts of all lymphocytes (B and T cells) in both FPIAP groups (T0 and Tres) except for a lower number of RTE cells, a higher T-CD4 memory cell count, increased numbers of regulatory B cells and a higher percentage of regulatory T cells (*p* < 0.01) in patients with acute FPIAP (T0) in contrast to the control group (Table 1). We analyzed the Treg (Foxp3)/TCD4+TemRA ratio, which reflects the capacity of Treg cells to control immune activation. This ratio was elevated in the FPIAP-T0 group compared to the control group (Figure 1).

**Figure 2:**
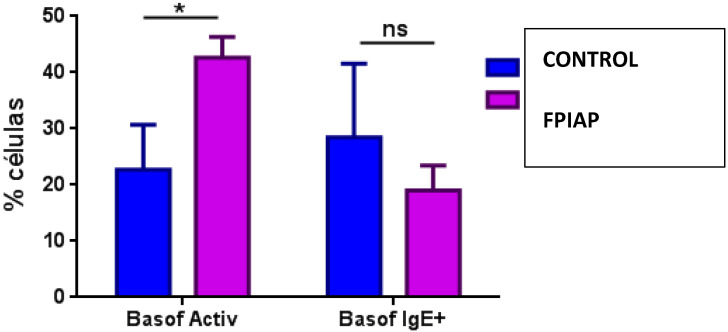
Comparison of percentage of activated basophils in FPIAP and control groups. *: statistically significant; ns: not statistically significant; Basof activ: activated basophils; Basof IgE+: IgE+ basophils.

In the analysis of myeloid cell populations, the levels of granulocytes (mainly eosinophils), dendritic cells (mDC2) and NK16+56- cells were significantly higher in patients with FPIAP compared to the control group (Table 2). The percentage of active basophils (not IgE+) was higher in the FPIAP group compared to the control group (Figure 2).

**Figure 3:**
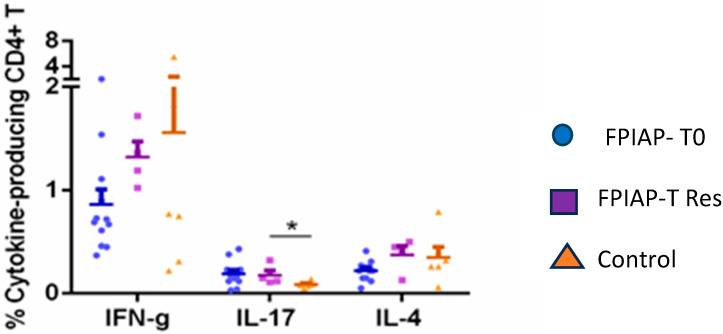
Comparison of percentage of cytokine-secreting CD4+ T cells in FPIAP T0, FPIAP Tres and control groups. *: statistically significant.

We compared the percentage of cytokine-secreting CD4+T cells in all the groups and found that the percentage of IL-17-secreting CD4+T cells was higher in the FPIAP groups (T0 and Tres) (Figure 3).

The immunophenotypic markers that showed a better ability to discriminate between control and FPIAP infants, according to the area under the ROC curves, were the absolute number of NK16+56- cells (AUC:0.916; *p* = 0.012;) and the number of granulocytes (AUC: 0.82; *p* = 0.006).

There were no differences in vitamin D values in the patients with FPIAP compared to control group of the same age (18.8 (15.4–24.32) µg/L vs. 15.7 (10.2–18.8); *p* = 0.208).

## 4. Discussion

The absence of differences in T cell populations between the FPIAP patients and healthy controls reveals a low level of participation of adaptive immunity in FPIAP. A lower count of RTE cells in the FPIAP groups could reflect an active differentiation of T cells from RTE cells into mature cells due to the inflammatory process. The higher count of central memory T cells suggests a recent activation of the immune system in the acute (T0) phase, which normalizes after resolution of the inflammation. In previous studies, non-IgE-mediated allergies were found to be mediated by T cells [22,26]. Later studies in infants with FPIES did not show a T cell-mediated physiopathology to explain this non-IgE-mediated allergy, at least not in the peripheral blood [27]. Therefore, other studies questioned the involvement of T cells in these allergies and believed that there was innate immunity participation, especially granulocytes and eosinophils, as was shown in our study [6,26].

Recent studies correlated intestinal microbiota with the innate immune system and identified dysbiotic features in this immune system in children with FPIAP [28]. Our study confirmed the theory that children with FPIAP have an altered innate immunity, which explains why their allergies improved after changing their intestinal microbiota [21]. We found no differences in the FPIAP patients that received different types of hydrolyzed formula (with or without probiotics), probably due to the small sample size.

The natural history of the patients included in our study is similar to the Italian cohort in [20]. The median age of tolerance was 10 months compared to 8 months for the Italian cohort, but a later prospective observational study cohort achieved tolerance at 6 months [21]. This was probably because in 10 patients in our study, the reintroduction of cow milk protein was delayed because of other viral infections (acute gastroenteritis).

We did not find a deficiency of Treg cells to explain the pathophysiology of allergic FPIAP, in contrast to previous studies on IgE-mediated cow’s milk allergy [29,30,31,32]. In fact, Treg cell counts were higher in the acute phase of inflammation and with an elevated Treg (Foxp3)/TCD4+TemRA ratio, which indicates the proper functioning of these Treg cells. Therefore, the role of Treg cells in FPIAP is controlling the immune response to the inflammation caused by cow’s milk protein. This is the first study on Treg cells in FPIAP that analyzed non-cryopreserved samples. There are two other studies with different methodologies that analyzed Treg cells in non-IgE-mediated allergies. The first one is Karlsson et al.’s study [33] that showed a higher number of Treg cells in infants who acquired tolerance after an FPIES reaction. The second one is the Cseh et al. study in which a lower percentage of Treg cells was found in patients with persistent FPIAP who needed elemental amino-acid formula to achieve resolution of the rectal bleeding, which is uncommon in FPIAP [34].

Therefore, allergic FPIAP does not appear to have an immune mechanism mediated by T cells, but it may be associated with innate immunity responses characterized by an increase in NK16+56- cells, eosinophils and dendritic cells. These cells could be evaluated in future studies as possible markers of non-IgE-mediated cow’s milk protein allergy. Eosinophils are the main cells that have been associated with these allergies, as they are found in cryptic abscesses in intestinal biopsies from non-IgE allergy patients [35]. One possible marker could be fecal eosinophil-derived neurotoxin, which was found to be elevated in infants with FPIAP [36]. Another possible marker to verify the participation of neutrophils in this reaction is fecal calprotectin, a cytosolic protein in neutrophils, monocytes and macrophages, which was also found to be elevated in those with non-IgE-mediated cow’s milk allergy compared to healthy infants [37,38]. An early recognition of this allergy can impact on the disease course, so fecal markers could be an important tool to avoid multiple food allergies or the risk of developing the allergic march [19].

Dendritic cells could be another possible marker of the FPIAP reaction, but they have not been studied before in non-IgE-mediated CMPA. Other antigen-presenting cells (APCs) have been studied recently. Macrophages have been associated with intestinal inflammation in FPIAP. Children with FPIAP showed a lower level of the macrophage inflammation protein MIP3a/CCL20 compared with controls [39] and had high levels of pANCA [40].

The higher percentage of IL-17-secreting CD4+T cells in the FPIAP group may be associated with the pro-inflammatory process. IL-17 contributes to the recruitment of neutrophils in inflammation and stimulates the production of cytokines such as TNF, which increases the population of neutrophils and monocytes at the point of inflammation [41]. An increase in IL-17 levels in the fecal samples of non-IgE CMPA infants has been found. An imbalance of TNFα/TGFβ at the intestinal mucosa can alter the intestinal permeability in these infants. This could explain the physiopathology of non-IgE CMPA reactions, as was proposed by other authors [22]. The absence of differences in IL-4 levels and IFN-γ secreting T cells in this study could indicate that type 1 and 2 immune responses are not involved in the FPIAP reaction.

**Figure 4:**
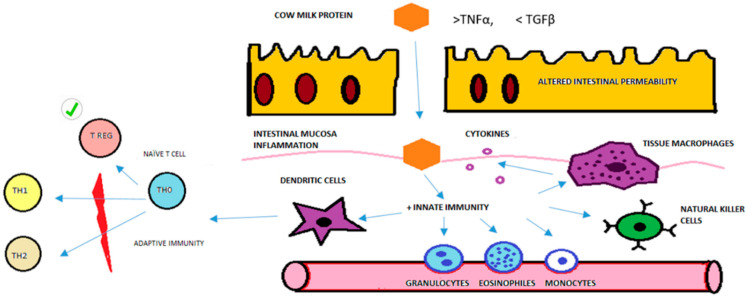
Model of physiopathological immune response and Treg cells in FPIAP based on the results of this study. Purple circle are cytokines. Red graphic is to explain that TH1 and TH2 immune response are not activated. Checkmark symbol is to highlight T Reg cells are normal in number and function in the physiopathology immune response of FPIAP.

In summary, we can conclude that Treg cells in children with FPIAP are normal and do not explain the pathophysiology of this allergy as they do in IgE-mediated allergies. There is a low level of participation of T cells in the physiopathology of the FPIAP reaction. We can speculate that the main problem in FPIAP is probably mediated by innate immunity, especially granulocytes such as eosinophils, NK cells, dendritic cells and other APCs (Figure 4). The altered intestinal permeability of FPIAP infants due to an imbalance of TNFα/TGFβ could allow the entry of cow’s milk proteins (CMPs). These CMPs could therefore activate granulocytes and eosinophils in the peripheral blood, which migrate to the rectal mucosa and accumulate in cryptic abscesses. The dendritic cells are then activated by inflammation and present antigens to naïve T cells to produce memory and effector cells. Then, the number of Treg cells would increase and limit the immune response so that type 1 or 2 immune response are not activated. This could explain the absence of specific antibodies and the low level of T cell participation in non-IgE-mediated CMPA that was found in this study and in previous studies [22].

There are some limitations in this study. Firstly, we could not compare Treg cells in both phases in this study (acute and resolution phases) because of the ethical limitation of taking blood samples twice from an infant. Secondly, we only studied peripheral blood and we did not study the intestinal mucosa so there could be T cell participation in that tissue, for example, intraepithelial lymphocytes. There is no need to take intestinal biopsies to diagnose FPIAP in an infant, so this study could not be performed.

## 5. Key Message

In this study, there was no deficiency of Treg cells to explain the pathophysiology of FPIAP, in contrast to the findings of previous studies on IgE-mediated cow’s milk allergy.

There is a low level of participation of T cells, in either type 1 or 2 immune responses, in the physiopathology of FPIAP.

The most important cells that induce the inflammation of the rectal mucosa are natural killer cells, eosinophils and dendritic cells. This suggests the participation of innate immunity in the FPIAP reaction rather than a T cell-mediated reaction.

Future investigations on non-IgE-mediated CMPA markers should focus on neutrophils, eosinophils or dendritic cells rather than T cells.

## Figures and Tables

**Table 1:** Comparison of lymphocyte counts in children with FPIAP (*T0* and *Tres*) and control group.

Immune Parameter (%)	FPIAP	FPIAP	CONTROL	*p*
	*T*0 (*n* = 22)	*Tres* (*n* = 10)	(*n* = 10)	
**Total lymphocyte count (CD3+)**	61.27 ± 8.4	61.7 ± 8.39	62.7 ± 7.44	0.85
**T-DP CD4+,CD8+**	**0.14 ± 0.19**	**0.07 ± 0.09**	**0.05 ± 0.07**	0.014 *
**TCD4+ cells**	46.22 ± 7.33	45.33 ± 8.06	47.72 ± 6.94	0.889
**CD4_38DR**	0.72 ± 0.35	0.72 ± 0.26	0.59 ± 0.25	0.388
**CD4_38**	82.33 ± 3.6	80.7 ± 5.67	82.66 ± 4.0	0.646
**-naïve**	85.89 ± 3.07	86.20 ± 4.90	87.19 ± 3.19	0.305
**-central memory (CM)**	10.68 ± 2.50	10.01 ± 4.35	9.08 ± 2.19	0.204
**-effector (TemRA)**	0.07 ± 0.06	0.08 ± 0.08	0.18 ± 0.22	0.345
**-activated (Act)**	0.54 ± 0.43	0.56 ± 0.36	0.51 ± 0.38	0.952
**-RTE**	**72.23 ± 4.98**	**72.76 ± 8.35**	**79.4 ± 5.58**	0.007 *
**-effector memory (EM)**	0.36 ± 0.22	0.43 ± 0.22	0.40 ± 0.47	0.251
**Treg cells**	7.6 ± 1.52	7.00 ± 1.52	7.49 ± 1.33	0.67
**Treg_38DR**	**4.89 ± 2.9**	**4.80 ± 2.88**	**1.6 ± 1.03**	0.003 *
**Treg_38**	55.53 ± 6.5	51.38 ± 5.33	56.86 ± 5.6	0.366
**-naïve**	69.75 ± 5.38	72.28 ± 8.56	73.83 ± 6.46	0.058
**-central memory (CM)**	**25.35 ± 4.99**	**22.47 ± 7.83**	**20.81 ± 5.35**	0.031 *
**-effector (TemRA)**	0.09 ± 0.06	0.07 ± 0.04	0.33 ± 0.58	0.345
**-activated (Act)**	2.96 ± 2.39	2.58 ± 1.18	2.76 ± 1.67	0.984
**-RTE**	**53.79 ± 4.08**	**55.87 ± 8.84**	**63.18 ± 8.04**	0.007 *
**-effector memory (EM)**	0.75 ± 0.36	0.94 ± 0.44	1.09 ± 1.19	0.857
**Treg Foxp3**	7.03 ± 1.39	5.89 ± 1.22	6.68 ± 1.43	0.588
**Foxp3 activated**	5.06 ± 3	4.7 ± 3	4.9 ± 3.3	0.887
**Foxp3_CD39**	1.8 ± 1.37	3.6 ± 3.3	3.6 ± 6.7	0.661
**Foxp3_memory**	41.23 ± 19.2	37.86 ± 17.24	33.62 ± 8.4	0.288
**Foxp3_naïve**	58.2 ± 19	61.38 ± 17	65.57 ± 8.1	0.288
**Foxp3_RTE**	**41.07 ± 14.2**	**44.94 ± 14**	**51.3 ± 7.6**	0.043 *
**TCD8+ cells**	13.88 ± 4.25	13.72 ± 4.53	12.8 ± 2.64	0.589
**-naïve**	88.74 ± 9.05	87.15 ± 10.82	87.16 ± 10.76	0.857
**-central memory (CM)**	7.78 ± 5.23	5.89 ± 3.10	7.68 ± 5.05	0.889
**-effector (TemRA)**	0.95 ± 2.41	3.78 ± 7.75	1.77 ± 2.75	0.388
**-activated (Act)**	1.07 ± 2.25	1.22 ± 2.31	1.38 ± 2.32	0.535
**-effector memory (EM)**	0.5 ± 1.6	0.7 ± 1.22	0.81 ± 1.59	0.562
**B CD19+ cells**	22.88 ± 7.21	21.04 ± 8.29	22.62 ± 9.17	0.826
**Naïve**	93.47 ± 2.06	92.34 ± 3.63	93.57 ± 1.53	0.92
**Memory switch**	0.87 ± 0.56	10.7 ± 0.63	0.75 ± 0.52	0.482
**Memory non-switch**	2.68 ± 1.41	2.13 ± 0.68	2.84 ± 1.34	0.704
**B regulatory (BR1)**	**13.08 ± 6.46**	**8.83 ± 2.51**	**7.19 ± 3.71**	0.010 *
**BR1 Real**	**0.09 ± 0.037**	**0.08 ± 0.04**	**0.04 ± 0.01**	0.002 *
**Breg I**	0.65 ± 0.25	0.83 ± 0.33	0.63 ± 0.20	0.974
**Breg II**	31.76 ± 7.24	27.23 ± 10	29.32 ± 8.9	0.671
**Plasmablasts**	0.71 ± 0.44	0.96 ± 0.94	0.68 ± 0.71	0.341

Variables with statistically significant differences are shown in bold and marked with *. T-DP: T cells double positive for CD4+ and CD8+; RTE: recent thymic emigrants; Breg I and Breg II: B regulatory cells type I and II.

**Table 2:** Comparison of myeloid cell populations in children with FPIAP (*T0* and *Tres*) and control group.

MYELOID CELL POPULATION (%)	FPIAP	FPIAP	CONTROLS	*p*
	*T*0 (*n* = 16)	Tres (*n* = 9)	(*n* = 7)	
**Granulocytes**	**26.92 ± 10.67**	**25.57 ± 8.84**	**17.8 ± 4.6**	**0.03** *
**Basophils**	0.80 ± 0.41	0.87 ± 0.33	0.85 ± 0.33	0.646
**Activated basophils**	36.13 ± 16.73	38.67 ± 21.14	30.43 ± 12.37	0.562
**IgE basophils**	13.37 ± 16.5	9.59 ± 8.9	13.3 ± 20	0.743
**Neutrophils**	**66.70 ± 19.44**	**71.42 ± 28.82**	**74.71 ± 7.51**	**0.013** *
**Eosinophils**	**30.22 ± 18.55**	**26.6 ± 28.87**	**21.94 ± 6.41**	**0.009** *
**Dendritic cells**	2.03 ± 0.78	1.65 ± 0.47	2.03 ± 0.71	0.891
**mDC2**	**6.28 ± 8.2**	**4.7 ± 6.66**	**0.7 ± 0.91**	**0.011** *
**mDC1**	16.90 ± 7.99	18.8 ± 5.44	18.9 ± 6.11	0.237
**-myeloid cells**	75.67 ± 11	73.1 ± 6.4	75.38 ± 8.68	0.680
**-plasma cells**	45.89 ± 19.7	42.85 ± 26.09	48.15 ± 19	0.731
**All NK cells**	21.38 ± 11.26	21.69 ± 13.42	18.73 ± 5.1	0.914
**NK16+56++**	12.56 ± 7.86	9.94 ± 4.2	13.03 ± 5.07	0.494
**NK16+56+**	**58.68 ± 12.1**	**67.7 ± 19.2**	**77.25 ± 6.1**	**0.001** *
**NK16+56-**	**24.8 ± 8.3**	**18.68 ± 12.4**	**8.67 ± 6.7**	**0.001** *
**Monocytes**	7.4 ± 2.4	6.83 ± 2.68	6.05 ± 1.16	0.261
**Classical monocytes**	87.26 ± 5.53	86.77 ± 8.4	90.68 ± 2.47	0.203

Variables with statistically significant differences are shown in bold and are marked with *. mDCs: myeloid dendritic cells; NK cells: natural killer cells.

## Data Availability

The original contributions presented in this study are included in the article. Further inquiries can be directed to the corresponding author.
